# Impairment of the DeISGylation Activity of Foot-and-Mouth Disease Virus Lpro Causes Attenuation *In Vitro* and *In Vivo*

**DOI:** 10.1128/JVI.00341-20

**Published:** 2020-06-16

**Authors:** Gisselle N. Medina, Paul Azzinaro, Elizabeth Ramirez-Medina, Joseph Gutkoska, Ying Fang, Fayna Diaz-San Segundo, Teresa de los Santos

**Affiliations:** a Plum Island Animal Disease Center (PIADC), ARS, USDA, Greenport, New York, USA; b Department of Pathobiology and Veterinary Science, University of Connecticut, Storrs, Connecticut, USA; c Kansas State University College of Veterinary Medicine, Manhattan, Kansas, USA; Instituto de Biotecnologia/UNAM

**Keywords:** ISG15, deISGylation, ISGylation, PLP, deubiquitination, foot-and-mouth disease virus, interferons, leader, picornavirus, proteases

## Abstract

Foot-and-mouth disease virus (FMDV) leader proteinase (Lpro) affects several pathways of the host innate immune response. Previous studies in bovine cells demonstrated that deletions (leaderless [LLV]) or point mutations in Lpro result in increased expression of interferon (IFN) and IFN-stimulated genes (ISGs), including, among others, the ubiquitin-like protein modifier ISG15 and the ubiquitin specific peptidase USP18. In addition to its conventional papain-like protease activity, Lpro acts as a deubiquitinase (DUB) and deISGylase. In this study, we identified a conserved residue in Lpro that is involved in its interaction with ISG15. Mutation W105A rendered *Escherichia coli*-expressed Lpro unable to cleave the synthetic substrate pro-ISG15 while preserving cellular eIF4G cleavage. Interestingly, mutant FMDV W105A was viable. Overexpression of ISG15 and the ISGylation machinery in porcine cells resulted in moderate inhibition of FMDV replication, along with a decrease of the overall state of ISGylation in wild-type (WT)-infected cells. In contrast, reduced deISGylation was observed upon infection with W105A and leaderless virus. Reduction in the levels of deubiquitination was also observed in cells infected with the FMDV LproW105A mutant. Surprisingly, similarly to WT, infection with W105A inhibited IFN/ISG expression despite displaying an attenuated phenotype *in vivo* in mice. Altogether, our studies indicate that abolishing/reducing the deISGylase/DUB activity of Lpro causes viral attenuation independently of its ability to block the expression of IFN and ISG mRNA. Furthermore, our studies highlight the potential of ISG15 to be developed as a novel biotherapeutic molecule against FMD.

**IMPORTANCE** In this study, we identified an aromatic hydrophobic residue in foot-and-mouth disease virus (FMDV) leader proteinase (Lpro) (W105) that is involved in the interaction with ISG15. Mutation in Lpro W105 (A12-LproW105A) resulted in reduced deISGylation *in vitro* and in porcine-infected cells. Impaired deISGylase activity correlated with viral attenuation *in vitro* and *in vivo* and did not affect the ability of Lpro to block expression of type I interferon (IFN) and other IFN-stimulated genes. Moreover, overexpression of ISG15 resulted in the reduction of FMDV viral titers. Thus, our study highlights the potential use of Lpro mutants with modified deISGylase activity for development of live attenuated vaccine candidates, and ISG15 as a novel biotherapeutic against FMD.

## Abstract (precis)

In this study, we identified an aromatic hydrophobic residue in foot-and-mouth disease virus (FMDV) leader proteinase (Lpro) (W105) that is involved in the interaction with ISG15. Mutation in Lpro W105 (A12-LproW105A) resulted in reduced deISGylation *in vitro* and in porcine-infected cells. Impaired deISGylase activity correlated with viral attenuation *in vitro* and *in vivo* and did not affect the ability of Lpro to block expression of type I interferon (IFN) and other IFN-stimulated genes. Moreover, overexpression of ISG15 resulted in the reduction of FMDV viral titers. Thus, our study highlights the potential use of Lpro mutants with modified deISGylase activity for development of live attenuated vaccine candidates, and ISG15 as a novel biotherapeutic against FMD.

## INTRODUCTION

Foot-and-mouth disease virus (FMDV) is a member of the *Aphthovirus* genus within the *Picornaviridae* family, and it is the etiologic agent of FMD, a disease of cloven-hoofed animals (1). The virus contains a single-stranded, positive-sense RNA genome of approximately 8,500 nucleotides surrounded by a nonenveloped icosahedral capsid. FMDV is genetically highly variable, and as such, it displays seven distinct serotypes, namely A, Asia-1, C, O, and Southern African Territories 1 to 3 (SAT 1 to 3), and numerous subtypes. Upon infection, the virus spreads very rapidly, usually achieving 100% morbidity. Strict trading policies and use of an effective inactivated virus vaccine has helped eradicate the disease from many countries; however, FMD remains endemic in most of the world, preventing the development of regions that rely on agriculture for subsistence. In parallel, occasional outbreaks in previously declared FMD-free regions may cause economic devastation (2). There is a need for novel preventive and therapeutic strategies for controlling this disease. Understanding virus-host interactions should help to identify novel cellular factors and mechanisms that participate in antiviral immunity against FMDV and could provide alternatives for therapeutic discovery.

During viral infection, expression of type I interferon (IFN) is induced, leading to the upregulation of IFN-stimulated genes (ISGs) which play a range of antiviral effector functions within the infected and neighboring cells (3). Regulation of IFN expression is the most essential target for viruses to evade and suppress innate immunity. We and others have shown that in the case of FMDV, downregulation of IFN and IFN-stimulated responses is mainly driven by the action of the viral leader protease (Lpro) (4). FMDV Lpro is a papain-like protease (PLP) known to block the cellular innate immune response, at both the transcriptional and translational level by utilizing different mechanisms, including (i) shutting down translation of host capped mRNAs through the cleavage of the translation initiation factor eIF4G (5, 6); (ii) downregulating IFN mRNA expression by causing degradation of NF-κB, IRF-3, IRF-7, and LGP2 (7–10); (iii) targeting the chromatin remodeling machinery to disrupt the expression of IFN and ISG mRNAs (11); and (iv) targeting of G3BP1/2 to block stress granule formation (12). It is important to note that other FMDV proteins have also been shown to negatively impact IFN and other cellular immune responses (4).

Ubiquitination is a posttranslational modification that plays a role at different points of the signaling cascade of innate immunity and involves the sequential reaction of three distinct types of enzymes, namely ubiquitin (Ub)-activating enzymes (E1s), Ub-conjugating enzymes (E2s), and Ub ligases (E3s). Similarly, the Ub-like (UBL) modifier ISG15 is conjugated to target proteins in a process known as ISGylation by the consecutive action of three enzymes that make up the ISGylation machinery (E1-Ube1L, E2-UbcH8, and E3-HERC5). However, unlike Ub, ISG15 and the ISGylation machinery are robustly induced by type I IFN (13) and can be upregulated upon viral infection (14).

Different receptors, adaptor proteins, and kinases are conjugated by Ub molecules to activate and transduce the downstream signaling for efficient production of the IFN, ISGs, and proinflammatory cytokines (15). In the case of ISG15, ISGylation can extend the activation state of certain signaling proteins, resulting in higher production of IFN and ISGs (16, 17). To regulate the overactivation of these pathways, cells express multiple enzymes capable of removing Ub or ISG15 from specific targets, and they are known as deubiquitinases (DUBs) and deISGylases (e.g., USP18). Similarly, viruses counteract induction of the antiviral immune response by reversing ubiquitination and ISGylation from host targets (18–20). In some cases, changes in viral pathogenesis have been observed by DUB/deISGylase gain of function due to viral recombination in natural environments (21). In particular for FMDV, it has been shown that overexpressed Lpro displays DUB activity, catalyzing the removal of ubiquitin from cellular substrates, including TRAF3, TRAF6, TBK, and RIG-I (22, 23), which are all mediators of the induction of IFN (4). Most recently, studies conducted in BHK-21 cells have demonstrated that during FMDV infection cellular proteins undergo deISGylation (24). Interestingly, the authors demonstrate that recombinant FMDV Lpro displays deISGylase activity on synthetic substrates; however, specific cellular host targets have thus far not been identified.

In order to elucidate intermolecular interactions between highly conserved amino acid residues in Lpro and ISG15, we conducted structural analysis followed by molecular modeling. Our analysis revealed the presence of an aromatic residue on Lpro, W105, that is required for optimal deISGylase activity in an *in vitro* cleavage assay. Importantly, engineering of an infectious clone carrying this mutation (LproW105A) rendered a viable FMDV with a perceptible level of attenuation and reduced deISGylation and DUB activity compared with wild-type (WT) virus during infection of porcine cells. Interestingly, reduced deISGylation and DUB function did not disrupt Lpro’s ability to block IFN and ISG expression during infection, although overexpression of ISG15 resulted in a significant reduction of FMDV replication. Most importantly, *in vivo* inoculation with FMDV W105A using an FMD mouse model (25) resulted in reduced lethality compared with inoculation with WT virus, suggesting that the inability to remove ISG15 confers viral attenuation *in vivo*. Our studies reveal that reducing/abolishing deISGylase activity in Lpro during infection renders the virus moderately attenuated independently of its ability to block the expression of type I IFN and other IFN-stimulated genes (ISGs).

## RESULTS

### Molecular modeling of Lpro in combination with ISG15.

**FIG 1:**
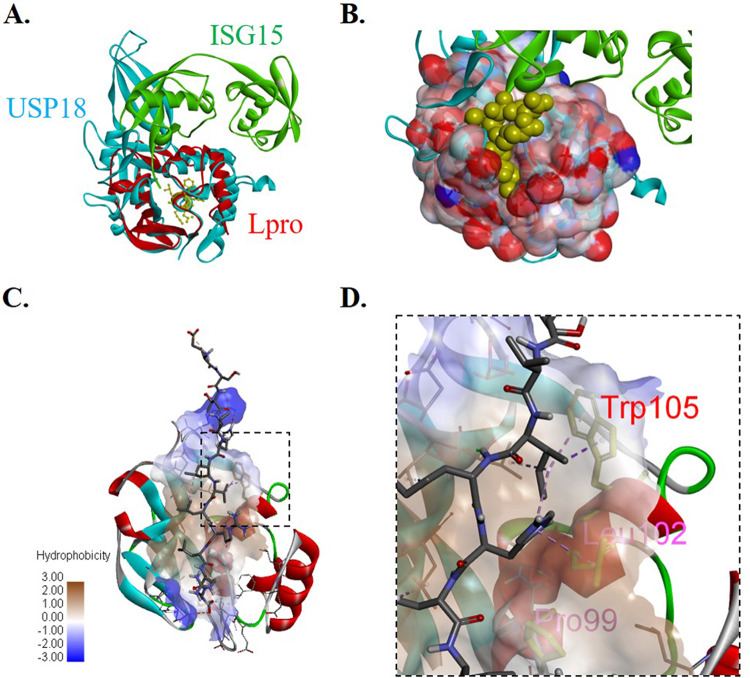
Structural analysis of FMDV Lpro and ISG15 binding interface. (A) Superimposition of Lpro (PDB number 1QBB) and USP18 cocrystalized with ISG15 (PDB number 5CHV) using catalytic residues as tether points highlights structural homology. (B) Simulated surface of Lpro shows potential binding cleft for ISG15 based on superimposition with USP18. (C) Image of peptide docking results showing putative binding mechanism between ISG15 C-terminal peptide and Lpro binding pocket rendered as ribbons. (D) Close-up interactions between aromatic amino acid Trp/W 105 in red and ISG15 C-terminal peptide, rendered as a stick. Other hydrophobic interactions are highlighted in magenta, namely Pro/P 99 and Leu/L 102. All images were rendered in Discovery Studio Visualizer.

In order to reexamine Lpro deISGylase function, we first investigated molecular homology through structural superimposition of Lpro with cellular murine deISGylase USP18 coupled with ISG15 (26) using catalytic residues as tether points (Fig. 1A). Superimposition of the two structures showed readily identifiable molecular homology between FMDV Lpro and the catalytic domain of the cellular deISGylase USP18 despite the lack of sequence homology (data not shown). A peptide generated from the C-terminal sequence of ISG15 was docked onto Lpro to generate a simulated structure for analysis. The docking simulation predicted the ligand bound in a binding pocket using several residues to coordinate the interaction (Fig. 1B). Analysis of the simulation revealed that ISG15 has similar predicted affinities for the binding pockets in Lpro or USP18, suggesting a possible interaction *in vivo*. Because ISG15 is not conserved among all species, our simulation was further refined using docking and molecular dynamics to include ISG15 ligands from bovine, porcine, murine, and human origin. By comparing interacting Lpro residues that are conserved across different FMDV serotypes (27) and that bind to ISG15 from multiple known host species, we identified FMDV Lpro residues that might be important for the interaction with ISG15. Consistent with previous studies that have examined the interaction between viral deISGylases and ISG15 (24, 28), the FMDV Lpro-ISG15 binding interface revealed a series of hydrophobic interactions in Lpro (Fig. 1C), including Trp/W105 and previously reported Pro/P99 and Leu/L102 (Fig. 1D) (24). We selected Trp/W105 for further characterization since it was the only aromatic residue conserved among all FMDV serotypes that presumably participates in hydrophobic ISG15 binding interactions.

### Tryptophan to alanine mutation in FMDV Lpro position 105 significantly reduces deISGylase function *in vitro*.

**FIG 2:**
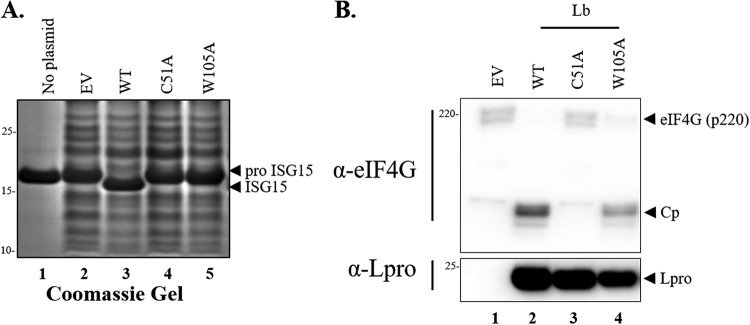
Lpro deISGylation *in vitro* activity. (A) Recombinant human proISG15 treated with bacterial extracts expressing WT or indicated mutant forms of Lpro. Lpro-dependent cleavage of proISG15 is detected as a visible 2-kDa band shift. (B) Immunoblot of samples treated with bacterial extracts expressing WT or Lpro mutants. Bacterial extracts prepared from IPTG-induced cultures were mixed with SK6 lysates, followed by incubation at 4°C to allow for Lpro-dependent enzymatic digestion of host proteins. Cleavage of eIF4G (220 kDa) generates a fragment of approximately 120 kDa (CP). For each figure, one representative blot is shown out of three independent experiments.

In order to examine whether the predicted aromatic residue in Lpro is involved in the interaction with ISG15, an *in vitro* cleavage assay was performed using a commercially available human ISG15 precursor protein (proISG15) as a substrate. Bacterial cell extracts were prepared from BL21(DE3) cells transformed with plasmid pET15-empty vector or pET15 encoding different versions of Lpro, namely wild type (WT), catalytically inactive C51A, and W105A, followed by incubation with either SK6 cell extracts or proISG15. As shown in Fig. 2A, cleavage of ISG15 was visualized as a protein band shift detected by Coomassie stain in only the cell extracts treated with bacterial extracts expressing WT Lpro (Fig. 2A, lane 3). In contrast, mutation of W105 in Lpro resulted in a significant reduction of proISG15 cleavage (Fig. 2A, lane 5), similarly to the catalytically inactive Lpro C51A (Fig. 2A, lane 4). It is important to note that all bacterial crude lysates derived from pET15-Lpro-transformed cells showed reactivity with our custom-made Lpro antibody (Fig. 2B, lanes 2 to 4). Interestingly, the W105A mutation minimally disrupted the ability of Lpro to cleave the cellular protein substrate eIF4G when bacterial extracts were incubated with whole SK6 lysates (Fig. 2B, lane 4). These results were consistent with Lpro-eIF-4G structural constraints previously reported (29). These data suggest that a separation-of-function mutation in Lpro (W105A) could be identified, albeit different incubation conditions were used to distinguish FMDV Lpro deISGylase from its canonical proteolytic activity on translation factor eIF-4G.

### Characterization of mutant Lpro W105A virus infectivity in different FMDV-permissive cell lines.

**FIG 3:**
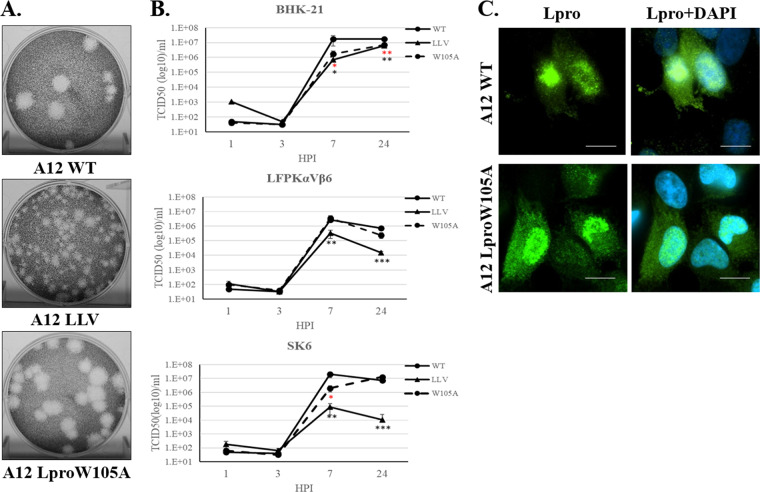
Plaque phenotype and viral growth kinetics. (A) Plaque phenotypes for FMDV A12 WT, A12 LLV, and A12 LproW105A were evaluated in BHK-21 cells. Cell monolayers were infected for 48 h in semisolid medium, followed by staining with crystal violet. (B) Kinetics of growth in multiple cell lines; BHK-21, LFPKαVβ6, and SK6 cells were infected at an MOI of 5 with WT, LLV, or LproW105A FMDV, and at the indicated times, virus titer was measured in BHK-21 cells. FMDV yield was determined by the endpoint dilution method on BHK-21 cells 24 h after infection. Titers are expressed as log_10_ 50% tissue culture infective dose (TCID_50_) per milliliter. The values are presented as the mean ± standard deviation of three independent experiments. (C) Fluorescent microscopy images from LFPKαVβ6 cells infected with WT or LproW105A FMDV at an MOI of 10 and fixed at 4 h postinfection. Samples were stained with anti-Lpro (green) and with nuclear stain 4′,6-diamidino-2-phenylindole (DAPI; blue). Scale bar represents 10 μm. Statistical analysis was performed using Student’s *t* test. *, *P < *0.05; **, *P* < 0.005; ***, *P* < 0.001; black asterisks represent statistical significance between LLV and WT; red asterisks represent statistical significance between W105A and WT.

To investigate the potential contribution of Lpro deISGylase activity in the context of a viral infection, we incorporated a W105A mutation in the FMDV serotype A12 infectious clone (30). Virus was recovered from cells electroporated with pRMC35-A12-LproW105A. Virus plaque assays were performed in BHK-21 cells to evaluate plaque morphologies of mutant A12-LproW105A compared with A12-LproWT and the attenuated virus A12-LLV (leaderless) (30). As shown in Fig. 3A, similar plaque sizes were detected for Lpro WT and W105A viruses. Interestingly, a small delay in the appearance of cytopathic effect (CPE) was observed for A12-LproW105A relative to A12-LproWT (data not shown). As previously reported, a relatively smaller plaque phenotype was displayed by mutant A12-LLV virus (31). Growth kinetics of A12-LproWT, A12-LLV, and A12-LproW105A were analyzed (Fig. 3B) in BHK-21, LFPKαVβ6, and SK6 cells. In all cell lines, A12-LproW105A replicated to levels comparable to those detected for A12-LproWT late in infection. However, a significant decrease in replication (∼10-fold) was detected at 7 h postinfection (hpi) in BHK-21 and SK6 cells infected with A12-LproW105A compared with WT virus. Further characterization of A12-Lpro W105A was determined by examining Lpro cellular distribution during infection since it has been previously described that WT Lpro can translocate to the nuclei of infected cells (8) and that mutations in other Lpro domains altered normal subcellular Lpro distribution (32). LFPKαVβ6 cells were infected with either A12-WT or A12-Lpro W105A FMDV and examined by immunofluorescence microscopy (Fig. 3C). Consistent with our previous studies (8, 11), indirect staining of Lpro using an anti-Lpro antibody showed similar nuclear and cytoplasmic signals for both A12-WT and A12-LproW105A FMDV-infected cells. These results indicated that the LproW105A mutation does not affect the normal pattern of Lpro cellular distribution during infection.

### LproW105A reduces deISGylation and deubiquitination activity in porcine cells during infection.

**FIG 4:**
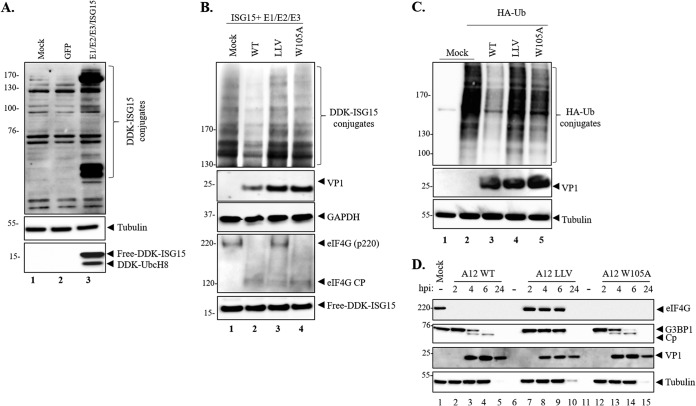
Lpro deISGylase activity during FMDV infection of porcine cells. (A) LFPKαVβ6 cells were mock transfected or transfected with either GFP or the ISG15 machinery (DDK-ISG15, E1 [UBE1L], E2 [UbcH8], and E3 [HERC5]). At 24 h posttransfection, proteins were resolved by SDS-PAGE and Western blot was applied to detect DDK-tagged free or conjugated ISG15 and UbcH8. (B) LFPKαVβ6 transfected as indicated in A were infected with FMDV A12WT, LLV, or LproW105A. At 6 hpi, cell lysates were prepared, and proteins were resolved in 3% to 8% SDS-PAGE, followed by Western blotting to detect DDK-tagged free or conjugated ISG15 and UbcH8, FMDV VP1, eIF4G, and GAPDH. (C) DUB activity of A12-WT, A12-LLV, and A12 Lpro W105A virus. LFPKαVβ6 cells were transfected with an HA-Ub expression plasmid, and 24 h posttransfection, cells were infected with the indicated viruses. Cells were lysed 4.5 h postinfection and analyzed by Western blotting to detect HA-tagged Ub conjugates, FMDV VP1, and tubulin. (D) LFPKαVβ6 cells were mock infected or infected with either A12-WT, A12-LLV, or A12-LproW105A virus, and lysates were collected at 2, 4, 6, and 24 hpi. Proteins were resolved by SDS-PAGE, and Western blot was applied to detect FMDV VP1, eIF4G, G3BP1, and tubulin. For each figure, one representative blot is shown out of three independent experiments.

To further investigate the function of Lpro deISGylation during FMDV infection, we first examined ISGylation in porcine cells susceptible to FMDV infection by mimicking activation of the ISG15 pathway without IFN stimulation. Transfection of LFPKαVβ6 cells with plasmids encoding DDK-tagged ISG15 and the ISG15 conjugation machinery (UBE1L, UbcH8, and HERC5) was performed and analyzed by Western blotting 24 h posttransfection (hpt). As shown in Fig. 4A, the anti-DDK (anti-Flag) antibody recognized N-terminally tagged ISG15 in the lysate prepared from DDK-ISG15-transfected cells (Fig. 4A, lane 3, bottom panel) and not in the cells transfected with the plasmid control encoding GFP or mock-transfected samples. The specific detection of ISG15 conjugates was observed by 24 hpt (lane 3) as multiple distinct bands, indicating that ISGylation had occurred. Detection of tubulin served as a sample loading control. To determine whether mutation W105A affected the ability of the virus to remove ISG15 conjugates from cellular target proteins, ISG15 conjugation machinery-transfected cells were infected with A12-WT, A12-LLV, or A12-LproW105A viruses and whole-cell lysates were collected at 6 hpi for Western blotting (Fig. 4B). It is important to note that a different gel system (3% to 8%) was needed to better assess ISG15 conjugation of relatively high-molecular-weight abundant target proteins. Robust detection of ISG15-modified protein substrates was observed in mock or infected cells that had been previously transfected with the ISG15 conjugation machinery (Fig. 4B). By 6 hpi, reduced ISG15 conjugation was clearly detected in the cell lysates derived from A12-WT-infected cells (Fig. 4B lane 2). In contrast, the levels of ISG15 conjugation were similar in the lysates of mock-, A12-LLV-, and A12-LproW105A-infected cells (Fig. 4B, lanes 1, 3, and 4). Interestingly, an increased DDK-ISGylation signal was detected in cells infected with leaderless virus (A12-LLV) (Fig. 4B, lane 3), consistently with the induction of a stronger host antiviral response. Canonical eIF4G cleavage was observed in A12-LproW105A and in A12 WT but not in A12-LLV-infected cells (Fig. 4B, bottom panels), regardless of decreased levels of ISGylated proteins. These results indicate that FMDV Lpro exhibits deISGylating activity in porcine cells and that mutation in LproW105A impacts optimal ISG15 cleavage during infection.

To determine whether a mutation in Lpro also disrupts its ability to remove Ub moieties from cellular substrates, we assayed Lpro W105A DUB activity by transfecting porcine LFPKαVβ6 cells with a plasmid encoding hemagglutinin (HA)-tagged Ub, followed by infection with A12-WT, A12-LLV, or A12-Lpro W105A viruses (Fig. 4C). As expected, infection of A12-WT virus strongly decreased the level of HA-tagged Ub conjugates (Fig. 4C, lane 3), consistent with its previously reported DUB function (22). DUB function was disrupted upon infection with either A12-LproW105A or A12-LLV virus (Fig. 4C, lanes 4 and 5). Taken together, our data indicate that W105 is required for efficient deconjugation of ISG15 and Ub from target cellular proteins during FMDV infection.

In order to rule out whether impaired deISGylation/deubiquitination in the Lpro mutation is due to its inability to process other cellular factors involved in innate immunity signaling, we examined the stress granule protein G3BP1. As previously shown (12), cleavage of G3BP1 was detected in WT-infected cell lysates from LFPKαvβ6 cells at 4 and 6 hpi (Fig. 4D, lanes 3 and 4). Similar patterns were observed in A12-LproW105A-infected cells (Fig. 4D, lanes 13 and 14), while a lack of G3BP1 cleavage was observed in A12-LLV-infected cell lysates (Fig. 4D, lanes 8 and 9). Taken together, these results indicate that the ability of A12-LproW105A to cleave G3BP1 is not affected, despite the observed block in deISGylation and deubiquitination activities.

### ISGylation inhibits FMDV replication in porcine cells.

**FIG 5:**
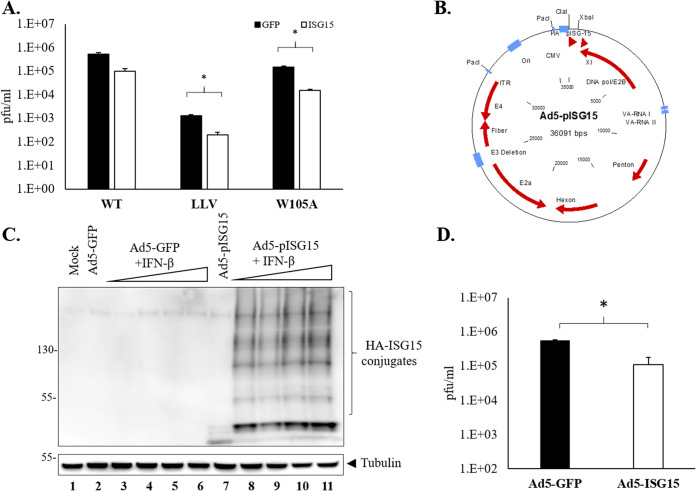
FMDV replication in cells overexpressing ISG15. (A) Viral titers were examined in LFPKαVβ6 previously transfected with ISG15 and infected at an MOI of 0.1 with WT, LLV, or LproW105A. FMDV yield was determined by plaque assay in BHK-21 cells. Plaques were counted 24 hpi, and titers were expressed as plaque forming units (PFUs) per ml. Values are presented as the mean ± standard deviation from three independent experiments. (B) Schematic representation of the replication-defective Ad5-pISG15 plasmid. HA-tagged pISG15 was cloned in the Ad5-Blue plasmid using ClaI and XbaI restriction enzyme sites. (C) Western blot analysis of protein ISGylation in mock or Ad5-GFP/Ad5-HA-pISG15-transduced LFPKαVβ6 cells in the presence or absence of increasing amounts of porcine IFN-β (5 to 40 U). Eighteen hours after IFN-β treatment, protein ISGylation was detected by using anti-HA antibodies. (D) Viral titers were examined in LFPKαVβ6 previously transduced with Ad5-HA-pISG15 and infected at an MOI of 0.1 with WT virus. Virus yield were determined by plaque assay in BHK-21 cells. The values are presented as the mean ± standard deviation of three independent experiments. Statistical analysis was performed using Student’s *t* test. *, *P < *0.05.

Distinct levels of antiviral activity mediated by ISG15 have been reported against different RNA viruses (33). In order to determine whether ISG15 can function as an antiviral agent against FMDV, LFPKαVβ6 cells were transfected with either GFP or ISG15 and ISG15 conjugation machinery, and at 24 h, cells were infected with either FMDV A12 WT, LLV, or LproW105A virus. As a control, cell lysates were evaluated by Western blot analysis for the presence of ISG15 and ISGylated proteins prior to infection (data not shown). A significant reduction in viral titers was observed when cells transfected with plasmids encoding ISG15 and the ISG15 conjugation machinery were infected with LLV or LproW105A (Fig. 5A). No reduction was detected in cells transfected with plasmid GFP control. Reduction in titer was also observed in WT-infected cells, but the difference was not statistically significant. To confirm these results using the FMDV host-specific ISGylation machinery during infection, we cloned the porcine ISG15 in a replication-defective human adenovirus type 5 (Ad5) vector (Fig. 5B) and examined its antiviral activity in swine cells. Transduction of LFPKαVβ6 cells with either Ad5-GFP or Ad5-pISG15 was performed, and detection of free-ISG15 and ISG15-conjugated proteins was examined by Western blot analysis (Fig. 5). To confirm that Ad5-pISG15 was functional under our experimental conditions, LFPKαVβ6 cells were treated with increasing amounts of IFN-β. As seen in Fig. 5C, ISG15-modified proteins were detected only in samples that had been treated with IFN-β and transduced with Ad5-pISG15 (lanes 8 to 11), indicating that Ad5-overexpressed porcine ISG15 induced the cellular ISGylation machinery required for ISG15 conjugation. A lack of differential dose response suggested that the system was saturated at the lowest IFN dose used (5 U/ml). A statistically significant reduction of endpoint titers was detected in cells transduced with Ad5-pISG15, but not with Ad5-GFP, after infection with WT FMDV (Fig. 5D). These results suggested that overexpression of ISG15 induced antiviral activity in porcine cells. All together, these data indicate that ISG15 has an inhibitory effect in the replication of FMDV.

### FMDV inhibition of IFN-stimulated gene expression is not affected by reduced Lpro deISGylase activity.

**FIG 6:**
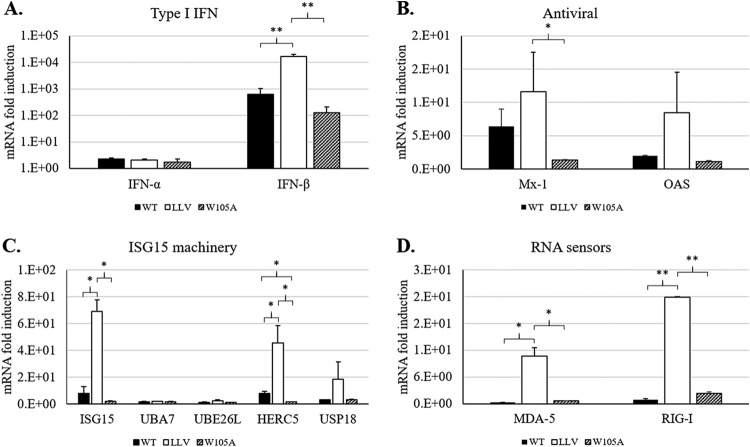
Expression of IFN-stimulated genes in response to WT, LLV, and W105 FMDV infection. Porcine SK6 cells were infected at an MOI of 0.1 with A12 WT, A12 LLV, or A12 LproW105A virus. Total RNA was isolated at 6 hpi, and different set of genes involved in type I IFN (A), antiviral activity (B), ISG15 machinery (C), and RNA sensing (D) were analyzed by qPCR using specific primers. Porcine GAPDH was used as an internal control. The results are expressed as the relative fold increase in gene expression with respect to mock cells. Results represent the mean from three independent experiments. Statistical analysis was performed using Student’s *t* test. *, *P < *0.05; **, *P* < 0.005.

Previous studies in bovine cells had demonstrated that infection with WT compared with LLV FMDV results in a lower expression of transcripts of genes involved in innate immunity, including those that participate in ISGylation (34). In order to determine whether reduced affinity of Lpro for ISG15 affects the production of IFN and ISGs, expression of specific mRNAs was analyzed by reverse transcription-quantitative PCR (qRT-PCR) during viral infection. As seen in Fig. 6A, similarly to WT, a significant inhibition of IFN-β expression was observed upon infection with FMDV LproW105A. In contrast, as previously reported, upregulation of IFN-β expression showed the highest levels in A12-LLV cells (7). Similar to IFN-β expression, antiviral genes Mx-1 and OAS-1 (Fig. 6B); genes encoding the ISGylation machinery, namely ISG15, HERC5, and USP18 (Fig. 6C); and RNA sensors MDA-5 and RIG-I (Fig. 6D) were upregulated to the highest levels only in LLV-infected porcine cells. These results indicate that disruption of Lpro deISGylase activity does not affect FMDV-dependent transcriptional downregulation of IFN-β, ISG antiviral responses, or components of the ISG15 pathway.

### *In vivo* attenuation of FMDV LproW105A in mice.

**FIG 7:**
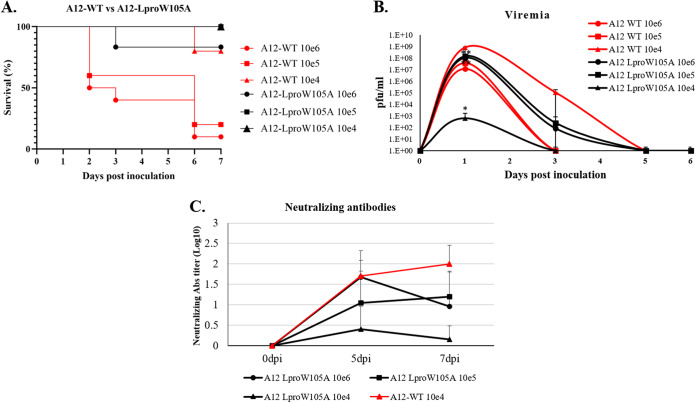
Virulence of A12-LproW105A in infected mice. Six- to 7-week-old female C57BL/6 mice (*n* = 6/group) were subcutaneously inoculated in the footpad with the indicated doses of A12-WT or A12-LproW105A mutant FMDV. (A) Clinical disease was followed for 7 days after inoculation, and percent survival was calculated as (number of surviving animals/number of animals per group) × 100, daily. (B) Serum samples collected at the indicated times after inoculation were assayed for the presence of virus by plaque assay on BHK-21 cells. (C) FMDV serum neutralizing antibody titers were determined by endpoint dilution and expressed as log TCID_50_/ml (48). Statistical analysis was performed using Student’s *t* test. *, *P* < 0.05; **, *P* < 0.005.

In order to understand whether the mutation W105A in FMDV Lpro causes attenuation *in vivo*, we examined this mutant in a mouse model of FMD (25, 35). Six- to 7-week-old C57BL/6 mice were inoculated with different doses of FMDV A12-LproW105A or A12-WT (6 mice/group) and checked for survival, viremia, and induction of neutralizing antibodies. As shown in Fig. 7A, mice inoculated with 10e6 or 10e5 PFU of A12-WT showed 10% and 20% survival, respectively, whereas mice inoculated with a lower dose (10e4 PFU) showed 80% survival. In contrast, 100% survival was observed in animals inoculated with 10e5 and 10e4 PFU of A12-LproW105A and 80% survival for the highest dose of A12-LproW105A (10e6 PFU). Viremia was detected for several days postinoculation (dpi), with a peak in the range of 10e7 to almost 10e9 PFU/ml at 1 dpi in mice inoculated with FMDV A12-WT (Fig. 7B). In all the groups inoculated with A12-LproW105A, the peak of viremia was also detected at 1 dpi. Animals inoculated with 10e4 or 10e6 PFU of A12-LproW105A had significantly lower titers (10e2 and 10e8 PFU/ml, respectively) than the titers measured in WT-inoculated animals. Interestingly, all animals inoculated with 10e5 PFU of A12-LproW105A survived despite the presence of high levels of viremia. In contrast, 80% of animals inoculated with the same dose of A12-WT virus died, even when the levels of viremia were lower than those detected in A12-LproW105A-inoculated mice. An analysis of humoral activity, in animals that survived the initial inoculation, showed detectable levels of neutralizing antibodies for both groups. However, animals inoculated with A12-LproW105A had lower levels of antibodies than those inoculated with similar doses of A12-WT virus (Fig. 7C). These results indicate that although FMDV A12-LproW105A is attenuated *in vivo*, it is still able to induce the production of neutralizing antibodies.

## DISCUSSION

In this study, by using molecular modeling, we identified a novel residue in FMDV Lpro required for optimal deISGylase activity. Our findings demonstrate, for the first time, that a viable FMDV with defective deISGylase activity and unaffected proteolytic activity on canonical cellular substrates, such as eIF4G, can be derived. Although other Lpro residues mediating its interaction with ISG15 have been recently identified (24), we propose that LproW105 is important for modulating viral infection kinetics. Furthermore, we show that Lpro mediates deISGylation during FMDV infection of natural host cells and that this function is important for optimal virus growth.

Reversing the posttranslational modification of cellular proteins conjugated to ISG15 to reduce the overactivation of innate immunity signaling pathways has been observed for other positive-sense, single-stranded RNA viruses that encode PLPs, including coronaviruses such as Middle East respiratory syndrome coronavirus (MERS-CoV), severe acute respiratory syndrome coronavirus (SARS-CoV-1 and SARS-CoV-2), and mouse hepatitis virus (MHV), and for the arterivirus porcine reproductive and respiratory syndrome virus (PRRSV). FMDV Lpro exhibits ISG15 binding profiles (hydrophobic interactions) similar to MERS-CoV and SARS-CoV deISGylases (28); however, ISG15-processing patterns induced by FMDV Lpro have been shown to be different (24). While most deISGylases cleave target proteins leaving a recyclable ISG15 product with a single G residue at the C terminus, FMDV Lpro deISGylase activity is characterized by leaving behind a G-G di-peptide signature on processed host proteins and no recyclable ISG15. This may pertain to the differential tropism of these viruses and the fact that ISG15 is not conserved across species (19). In this study, we considered the diversity of ISG15 for multiple FMDV host species and highlighted unique features of FMDV Lpro for substrate recognition.

In addition to the role in deISGylating events, Lpro is known to have DUB activity (22); however, it is not clear what are the molecular sites that determine the cross-specificity for Lpro binding to Ub versus ISG15. It is important to mention that, thus far, all studies on these Lpro functions have been performed with overexpressed Lpro and candidate target host proteins (22). In this study, we demonstrated that infection with A12-Lpro W105A also disrupted efficient DUB activity (Fig. 4C), suggesting that Ub and ISG15 may have overlapping target-binding profiles. Understanding the specific FMDV Lpro determinants for binding Ub may highlight the relative differential contribution of ubiquitination and ISGylation to the antiviral signaling pathways induced upon FMDV infection.

Most importantly, our studies show that FMDV Lpro deISGylase activity is not critical for effective type I IFN antagonism despite its known ability to block downstream IFN signaling effectors as a consequence of DUB function (22). Interestingly, examination of mRNA transcripts involved in ISG15 conjugation resulted in a significant reduction of the ligase HERC5 in cells infected with FMDV LproW105A compared to WT-infected cells (Fig. 6C). Since HERC5 can regulate the conjugation of ISG15 to different cellular targets, its downregulation may favor a greater availability of unconjugated ISG15. Thus, free forms of ISG15 could modulate other novel antiviral functions, contributing to the observed delay in viral replication (Fig. 3B). In WT-infected cells, modified cleavage of ISG15 (24) results in nonfunctional ISG15 molecules that cannot be recycled for additional rounds of ISGylation or perhaps display other unrecognized antiviral functions. It is also possible that Lpro deISGylation activity is important for modulating IFN-independent antiviral pathways that have not been explored in our studies. In fact, upregulation of ISG15 transcripts has been detected in porcine cells treated with poly:IC without the requirement of IFN production (36). Similar results have been observed with other viruses, such as cytomegalovirus (CMV) (37). Identification of ISGylated substrates during FMDV infection should illustrate new functions of Lpro.

Although ISG15 has been shown to have broad antiviral activity against different RNA viruses, to date, no such information is available for FMDV. In this study, we show that overexpression of ISG15 affects virus replication in porcine cells. Most importantly, FMDV with reduced (A12-LproW105A) or a complete loss of deISGylase function (A12-LLV) was more sensitive to the ISG15-induced antiviral effects. However, the mechanism of antiviral function against FMDV remains elusive. It is possible that FMDV proteins may be substrates for the ISG15 conjugation machinery due to its known ability to target *de novo* protein synthesis (38). In fact, previous reports have shown that the viral 2A protease of coxsackievirus (CVB3) can be ISGylated, thus preventing eIF4G cleavage during infection of HeLa cells (39). ISG15 modifications of other viral proteins have been reported for influenza virus, human CMV, and human papillomavirus (19). Additionally, ISGylation has been related to the release of virus particles that require the endosomal sorting complex required for transport (ESCRT) pathway, such as HIV-1, Ebola virus (EBOV), and avian sarcoma leukosis virus (ASLV) (33). Although the involvement of this pathway is not associated with FMDV release, it has been recently reported that nanovesicles may play a role in transmission of FMDV *in vitro* and *in vivo* (40). Furthermore, it is known that exosome release could be modulated by the expression of ISG15, presumably affecting related biological functions (41). It will be interesting to determine whether FMDV proteins are targets for ISGylation during infection and which are the host substrates for Lpro deISGylase activity. To this end, our results demonstrate that Lpro deISGylation activity is required for optimal FMDV growth but not as the result of impairing the induction of IFN and ISGs. Moreover, we demonstrate that impairment of this activity results in viruses that are attenuated not only in tissue culture but also *in vivo* in mice. Although mice are not the natural hosts for FMDV, our previous studies using this model have always shown a pattern that was later reproduced in natural host species (35, 42). Further evaluation in livestock species is warranted for mutant FMDVs with impaired deISGylase activity. Importantly, the discovery of mutations that are tolerated by the virus opens a venue for the rational design of novel modified live or inactivated FMD vaccines that hopefully will contribute to the effective control of such an important transboundary animal disease.

## MATERIALS AND METHODS

### Molecular modeling.

The open-source program for doing molecular docking autodock Vina (43) was utilized, and a peptide of the 13 C-terminal residues of ISG15 was used to identify the putative binding pocket on Lpro (PDB number 4QBB). Molecular dynamics was performed using GROMACS (44). Simulations were executed to assess the ability of different peptides to bind to different iterations of Lpro as well as USP18-mISG15 (PDB number 5CHV) (26) as a control for deISGylase.

### Plasmids and reagents.

The gene Lpro was cloned in plasmid pET15b-6X-His (Novagen, Billerica, MA) using the restriction sites NdeI and BamHI. Site-directed mutagenesis at the active site of Lpro was used to generate a proteolytically inactive protein (LproC51A). Plasmid pCMV-ISG15 encoding human ISG15 N-terminally tagged with DDK (Flag) and plasmids encoding untagged proteins of the ISG15 conjugation machinery (UBE1L and UBCH8) were obtained from the Krug Lab (University of Florida, Gainsville, FL) and have been described previously (45). Human ubiquitin (GenBank accession number AB089617; Genescript, Piscataway, NJ) was cloned into the mammalian expression vector pCI using NotI and NheI restriction sites. A hemagglutinin (HA) tag was incorporated in-frame at the 5′ end of the ubiquitin gene. A replication-incompetent human Ad5 vector encoding porcine ISG15 was constructed by digestion of the pAd5Blue vector with XbaI and ClaI restriction enzymes (46) and ligation with a synthetically made insert (Genescript) containing pISG15. The plasmid construct was confirmed by sequencing. For Western blot analysis, the following primary antibodies were used: monoclonal rabbit anti-DDK, mouse anti-DDK, and mouse anti-GAPDH from Origene (Rockville, MD); monoclonal mouse anti-HA from Sigma (St. Louis, MO); and monoclonal antibody (MAb) mouse anti-tubulin alpha from NeoMarkers (Fremont, CA), anti-eIF4G from Bethyl Laboratories (Montgomery, TX), and anti-G3BP1 from Aviva Systems Biology (Sand Diego, CA). Goat anti-mouse immunoglobulin G (IgG) and goat anti-rabbit IgG secondary antibodies conjugated to horseradish peroxidase (HRP) were obtained from Pierce (Rockford, IL). When indicated, protein samples were resolved by SDS-PAGE and detected by Western blotting (WB) using the specific antibodies and an ECL chemiluminescence kit (Bio-Rad, Hercules, CA). Images were acquired with the Azure Imager c300 digital imager.

### Cells.

Porcine kidney cells overexpressing the bovine integrin αVβ6 (LFPKαVβ6) or SK6 cells were obtained from APHIS/Foreign Animal Disease Diagnostic Laboratory (FADDL). These cells were maintained in minimal essential medium (MEM; Gibco BRL, Invitrogen, Carlsbad, CA) containing 10% fetal bovine serum (FBS) and supplemented with 1% antibiotics and nonessential amino acids. Human embryonic kidney 293 (HEK 293) cells lines (ATTC CRL-1573) from the American Type Culture Collection (ATCC; Rockville, MD) were used to propagate the Ad5-pISG15 virus. HEK 293 cells were maintained in MEM containing 10% FBS supplemented with 1% antibiotics (Gibco BRL), nonessential amino acids, and l-glutamine. Baby hamster kidney strain 21 (BHK-21; clone 13, ATCC CL10) cells were obtained from the ATCC were used to propagate virus stocks and to measure virus titers. BHK-21 cells were maintained in MEM containing 10% calf serum and 10% tryptose phosphate broth supplemented with 1% antibiotics and nonessential amino acids. Cell cultures were incubated at 37°C in 5% CO_2_.

### Viruses.

FMDV A12-WT (wild type) was generated from the full-length serotype A12 infectious clone pRMC35 (30), and A12-LLV2 (leaderless virus) was derived from pRMC35 by deletion of the Lb coding region (31). FMDV A12-LproW105A was constructed by site-directed mutagenesis using the QuikChange kit. All viruses were derived and propagated in BHK-21 cells, concentrated by polyethylene glycol precipitation, titrated on the same cells, and stored at –70°C. Viral full-length sequences were confirmed by DNA sequencing of derived viral cDNA using an ABI Prism 7000 instrument (Applied Biosystems, Thermo Fisher). The Ad5-pISG15 virus was derived and propagated in HEK 293 cells as previously described (47).

### Viral infections.

Cultured cell monolayers were infected with FMDV at the indicated multiplicity of infection (MOI) for 1 h at 37°C. After adsorption, cells were rinsed with 150 mM NaCl in 20 mM morpholino-ethane-sulfonic acid (MES) (pH 6.0) to remove noninternalized virus, and MEM containing nonessential amino acids, antibiotics, and antimycotic (Gibco BRL) was added, followed by incubation at 37°C. At specific times, infected cells were frozen and thawed followed by the determination of virus titers by endpoint dilution in BHK-21 cells (48).

### Lpro cleavage assays.

To determine the ability of Lpro mutants to cleave substrate pro-ISG15, WT and Lpro mutants were cloned in pET15b plasmids and transformed in BL21 Escherichia coli. Bacteria expressing the different Lpro variants were grown at 37°C in Luria-Bertani broth containing ampicillin for 16 h. Cultures were diluted 1:50 into 50 ml of the same medium and grown under similar conditions until the optical density at 600 nm (OD_600_) reached approximately 0.6 (∼2 h). Expression of Lpro was induced by adding 1 mM isopropyl-β-d-thiogalactopyranoside (IPTG) and continuing incubation for 2 h at 37°C. Bacterial cells were pelleted by centrifugation and lysed in 5 ml/g of Bug Buster protein extraction reagent (Sigma-Aldrich, Saint Louis, MO) containing 250 U/μl Benzonase (Sigma-Aldrich) on ice. Lysates were clarified by centrifugation, and aliquots of 5 μl of supernatant were assayed on 12.5 μg of purified human recombinant proISG15 (Boston Biochem, Boston, MA) or on crude protein lysates of SK6 cells. Reaction mixes were incubated at either room temperature for 1 h (pro-ISG15) or at 4°C for 18 h (SK6 crude lysates), at the end of which protein products were resolved by SDS-PAGE. Coomassie G-250 stain was used to visualize protein bands using the SimplyBlue SafeStain kit (Invitrogen, Carlsbad, CA), following the manufacturer’s directions. Western blotting was used to detect cleavage products in cellular SK6 extracts.

### Real-time RT-PCR.

Total RNA was isolated from mock-infected and infected LFPKαVβ6 or SK6 cells using a RNeasy minikit (Qiagen) following the manufacturer’s instructions. cDNA was synthesized from 1 μg of total RNA treated with DNase I (Sigma) using Moloney murine leukemia virus reverse transcriptase (Invitrogen) and random hexamers following the manufacturer’s directions. The relative expression of IFN-α, IFN-β, ISG-15, HERC5, USP18, UBA7, UBE26L, Mx-1, OAS-1, RIG-I, and MDA-5 on the total cDNA was determined by qPCR using the PerfeCTa SYBR green FastMix, carboxy-X-rhodamine (Quanta Biosciences, Gaithersburg, MD), and specific primers previously described (11). The expression of glyceraldehyde-3-phosphate dehydrogenase (GAPDH) was used as internal control. cDNA was amplified for 40 cycles with an ABI Prism 7500 sequence detection system (Applied Biosystems, UK). Relative gene expression was quantitated using the threshold cycle (2^−ΔΔ^*^CT^*) method.

### Mouse experiments.

Six- to 7-week-old female C57BL/6 mice were purchased from Jackson Laboratory (Bar Harbor, ME) and were acclimated for 1 week. Groups of 6 mice were anesthetized with isoflurane (Webster Veterinary, Devens, MA) and immediately infected subcutaneously in the left rear footpad with 10e4, 10e5, or 10e6 PFU of FMDV A12-WT or FMDV A12-LproW105A, in a volume of 50 μl. Animals were monitored daily for 7 days. Viremia and titers of neutralizing antibodies were determined by standard plaque assay or endpoint dilution on BHK-21 cells, as previously described (42).
